# Supplementary material for: Patterns of Intron Gain and Loss in Fungi
Source: PLoS Biol. 2004 Nov 30;2(12):e422. doi: 10.1371/journal.pbio.0020422 (PMC532390; doi:10.1371/journal.pbio.0020422)
Supplement: Table S1 — Also available at http://genes.mit.edu/NielsenEtAl/. (4.3 MB ZIP). [file pbio.0020422.st001.zip › NielsenEtAl/html/438.html]

AN7435.1.NCU07482.1.MG03632.1.FG00911.1


```
 CLUSTAL W (1.82) Multiple Sequence Alignments - Introns Inserted


Sequence 1: NCU07482.1	449 aa
Sequence 2: MG03632.1	395 aa
Sequence 3: FG00911.1	396 aa
Sequence 4: AN7435.1	374 aa
Alignment Length: 452 aa
Number Identitical Residues: 158 aa
Alignment Score (without introns) 7931


MG03632.1 	--MSTLKRKANG--PLSASDAKKPKQNASITSFFAAPK-------TTPASGATTSS----
NCU07482.1	--MSSLKRKAGSSQPGPSPDAKKPNQNGNIMSFFGVPKPGGPLNSSSPTTRSTSRSQSQL
FG00911.1 	--MSALKRKAGAS---AGNDSKKTKADGNIASFFAAAP--------KPAAGTVSAS----
AN7435.1  	MASPSLKRSAGDLAN-PAVESKRPKTNGSITSFFGAPK-------PKENGAATTKS----
          	 : .:***.*.     .. ::*:.: :..* ***...        ..    :.: *    

MG03632.1 	-SPSSTAAPAASAPAHPPAVS--FNKQKWVEKLTNEQRELLKLEIDTLDESWLALLKDEI
NCU07482.1	SNSNSSTTTTATTTPDPATVTKKFNKEKWVASLTPEQRDLLQLEIDTLDESWLAHLKEEI
FG00911.1 	-----ASAPAAR-----------FDKTKWVDSLKPEQKELLQLEIDTLDESWLAHLKDEL
AN7435.1  	-----AVPSSVSS----------FNKQKWVATLTPEQKELLRLEIDTMDESWLAHLKEEL
          	     : ..:. :          *:* *** .*. **::**:*****:****** **:*:

MG03632.1 	TTKEFLDLKRFLDREANAGKKVFPPREDVYSW2SRHTPFHTVKVVILGQ~DPYHNINQAH
NCU07482.1	VTKEFFDLKRFLKKEWET-KTIFPPKEDIYSW2SRHTPLPTVRVLILGQ~DPYHNHNQAH
FG00911.1 	VSKEFLDLKRFLDREITSGRKVFPPRDDIYSW2SRHTPFSNVKVVIVGQ~DPYHNDNQAH
AN7435.1  	IKPEFLALKRFLQKEKQMGVKVFPPEEDIYSW2SRHTPLHKVKVVVIGQ0DPYHNYNQAH
          	 . **: *****.:*   . .:***.:*:*** *****: .*:*:::** ***** ****

MG03632.1 	GLAFSVRPPTKAPPSLKNMYICLKNDYPEFTPPPNNSGLLTPWAQRGVLMLNTCLTVRAH
NCU07482.1	GLSFSVRPPTPAPPSLRNIFIGLARDYPSFTPPPSKSGLLTPWATRGVLLLNTCLTVRAH
FG00911.1 	GLAFSVRPPTPAPPSLKNMYIALKKDYPTFEPPANRGGLLTPWAERGVLMLNTCLTVRAH
AN7435.1  	GLAFSVRPPTAAPPSLVNIYASIKNDYPSFVPPPNKGGLLIPWAERGVLLLNTCLTVRAH
          	**:******* ***** *::  : .*** * **....*** *** ****:**********

MG03632.1 	EANSHANRGWEKFTQKIIELVAGRRSRGVVFMAWGSPAAKRVIKVDQKKHLVLKAVHPSP
NCU07482.1	EANSHANRGWERFTQKVIDLVNERQKKGVVFMAWGAPAGKRMAKVDGKRHLVLRSVHPSP
FG00911.1 	EANSHSNRGWERLTQKVIDIIAQKRTRGVVFMAWGTPAGKRVQKIDRVKHLVLQSVHPSP
AN7435.1  	QAASHSNKGWERLTQKAIDVVTRVRTRGVVFLAWGTPAGKRITGINRDRHCILQSVHPSP
          	:* **:*:***::*** *:::   :.:****:***:**.**:  ::  :* :*::*****

MG03632.1 	LSASRGF~FDCGHFRKANEWLVQRYGDGEEIDWSLTEGVSLLP-----------KPKVEV
NCU07482.1	LSAHRGF~FDAGHFKKGNEWLEGRYGKEGRVDWSLVEGKSIFADEDGEKGKTEGKGKGEG
FG00911.1 	LSASRGF~FDCNHFKKANDWLVSRYGPDGEVDWALGPGTSTKAPIK-ESDANEAKVAEKK
AN7435.1  	LSAHRGF0LNNQHFKKCNDWLAERYGPDEIIDWSLVS-----------------KQEASP
          	*** *** ::  **:* *:**  ***    :**:*                   *   . 

MG03632.1 	VKAAPEAK--KVKKDEEE----FD----FDEG--EELEALEELRKIEDNKKPE~VPAPLT
NCU07482.1	KKDGKDGKGEKEKKEEEEEEDEFEGGEIFDEEGLKEVEAVVEKVVTEEGGKEE~LKTDLM
FG00911.1 	AEAIEKSKPEAVKKVEENKENDVE----FDEDEEEALEEALRLAEEEEKEKKK2IRDLGM
AN7435.1  	ASAVVQSSSVTVNQSTQQA---------VTESANKRTVTTKSSTLPQEAEFEE~DLDALE
          	 .   ...    ::  ::          . *   :           ::    :       

MG03632.1 	AKPG~---VEVKGDENKNPSTATA-------------
NCU07482.1	VKKK~KNKGEEGGDENAIPVPEEEEEEEEQTEKMEED
FG00911.1 	KVQG1----YHMWRLNKIIVEEM--------------
AN7435.1  	ALAA~--------LENSQES-----------------
          	               *
```
